# Measles and Rubella Seroprevalence in Mother–Infant Pairs in Rural Nepal and the United States: Pre- and Post-Elimination Populations

**DOI:** 10.4269/ajtmh.17-0836

**Published:** 2018-09-17

**Authors:** Alastair F. Murray, Janet A. Englund, James M. Tielsch, Joanne Katz, Laxman Shrestha, Subarna K. Khatry, Kristen Carlin, Steven C. Leclerq, Mark C. Steinhoff, Helen Y. Chu

**Affiliations:** 1 George Washington University School of Medicine & Health Sciences, Washington, District of Columbia;; 2 Department of Pediatrics, Seattle Children’s Hospital, University of Washington, Seattle, Washington;; 3 Department of Global Health, Milken Institute School of Public Health, George Washington University, Washington, District of Columbia;; 4 Department of International Health, Johns Hopkins Bloomberg School of Public Health, Baltimore, Maryland;; 5 Department of Pediatrics and Child Health, Nepal Institute of Medicine, Kathmandu,; 6 Nepal Nutrition Intervention Project—Sarlahi (NNIPS), Kathmandu, Nepal;; 7 Children’s Core for Biomedical Statistics, Seattle Children’s Research Institute, Seattle, Washington;; 8 Global Health Center, Cincinnati Children’s Hospital Medical Center, Cincinnati, Ohio;; 9 Division of Allergy and Infectious Diseases, University of Washington, Seattle, Washington

## Abstract

We sought to compare seroprevalence of protective measles and rubella-specific antibody in mother–infant pairs across two populations: a pre-disease elimination Nepal population with recently introduced rubella vaccine and post-disease elimination U.S. population. Qualitative measles and rubella immunoglobulin G was assessed in maternal serum and cord blood from 258 pairs in Nepal, 2012–2013 and 49 pairs in Seattle, WA, 2014–2015. High rates of protective antibody were observed in both populations. Two hundred and forty-four (95%) pregnant women in Nepal had protective measles antibody versus 44 (92%) in Seattle (*P* = 0.42). Ninety-six percent of infants in Nepal (*N* = 246) and Seattle (*N* = 43) had protective measles antibody (*P* = 0.75). Ninety-four percentage of pregnant women in Nepal (*N* = 242) and Seattle (*N* = 45) had protective rubella antibody (*P* = 0.23). Two hundred and thirty-eight (93%) infants in Nepal had protective rubella antibody versus 44 (98%) in Seattle (*P* = 0.12). Continued surveillance will be necessary to ensure protective immunity, inform progress toward disease elimination in Nepal and avoid reemergence in the United States.

Measles and rubella are highly infectious vaccine-preventable viral diseases. Measles (rubeola), characterized by maculopapular eruptions, pneumonia, and diarrhea, is a leading cause of early childhood mortality worldwide.^1^ Rubella infection during pregnancy may lead to congenital rubella syndrome (CRS), characterized by sensorineural deafness and ophthalmic and cardiac abnormalities.

Maternally derived passive immunity against measles and rubella protects neonates from infection during the first months of life, when morbidity and mortality from these diseases is highest. Age at vaccine administration differs worldwide, balancing higher disease risk in younger infants with greater long-term protection and decreased vaccine failure when administered in older infants.^2,3^ These vaccines are contraindicated during pregnancy because of theoretical concern for fetal infection, although reports of measles–mumps–rubella (MMR) vaccine given inadvertently during pregnancy have not demonstrated safety signals.^4^ Following introduction of the MMR vaccine in the United States in 1971, reported cases of measles, mumps, rubella, and CRS decreased by 99%.^5^ There has been a rebound in U.S. measles cases with outbreaks in California (2014) and Minnesota (2017) and in 2014, a record number of annual cases (*N* = 667) in the post-elimination period.^6–8^

The World Health Organization (WHO) Global Vaccine Action Plan calls for 95% childhood coverage for two doses of measles and rubella vaccination in 47 priority countries with high disease burden by 2020.^9^ Monovalent measles vaccine became routine in Nepal, a priority country, in 1989. Routine combination of measles–rubella vaccination in Nepali children (9 months to 15 years) began in 2012–2013.^10^ A 2016 WHO survey of measles and rubella vaccine coverage in infants aged 12–23 months showed 83% coverage in Nepal and 92% U.S. coverage.^11^

Our study sought to compare seroprevalence of protective measles and rubella antibody in mother–infant pairs across two distinct populations: a population in Nepal, with established measles vaccination and recently introduced rubella vaccination, and in Seattle, WA, a post-measles and rubella elimination population with long established vaccination.

Although vaccine coverage data are readily available, there are limited data on population seroprevalence of measles and rubella antibodies, especially in low-resource settings. In a 2008 study of 2,224 Nepali women of childbearing age (15–39 years), 90.8% of women were rubella IgG seropositive from natural infection.^12^ Nepali women born before 1997 would not have received routine rubella vaccine coverage, and any immunity is from the history of natural infection. By comparison, estimated U.S. maternal rubella immunity during our study period is 93.7% using the corresponding age group from 1999 to 2004 National Health and Nutrition Examination Survey data.^13^ Measles seroprevalence data are unavailable for either population. We hypothesized high rates of measles and rubella immunity in the Seattle population and lower rates of measles immunity in Nepal based on WHO survey data and barriers to care in the low-resource setting and high rates of natural rubella immunity in mothers and infants in Nepal consistent with the previous pre-vaccine study.^12^

Maternal venous and infant cord blood samples were collected from mother–infant pairs at delivery in Seattle and Nepal. In Nepal, samples were collected from July 2011 to March 2014 as part of a randomized clinical trial of maternal influenza immunization. Verbal informed consent was obtained from women in Nepal using language approved by Institutional Review Boards of Cincinnati Children’s Hospital, Johns Hopkins Bloomberg School of Public Health, and Nepal Health Research Council with deferral from Seattle Children’s Hospital. The maternal influenza trial was registered at clinicaltrials.gov (NCT01034254). Women in the community were enrolled in their second trimester of pregnancy and infants were enrolled at birth.^14^ In Seattle, a prospective surveillance study of maternal transplacental antibody transfer in healthy pregnant women was conducted from December 2014 to September 2015. Written consent was obtained from participants with language approved by Seattle Children’s Hospital Institutional Review Board. Healthy pregnant women at least 18 years of age and at least 20 weeks’ gestation were enrolled at time of visit to a midwife clinic and infants were enrolled at birth. Exclusion criteria included underlying immunocompromising conditions, increased risk of preterm birth, systemic steroid use > 7 days, immunomodulating or investigational drugs or blood products during pregnancy.

Specimens were aliquoted at study sites and transported for storage at −20°C at the University of Washington, Seattle, WA. Differences in mother and infant denominators are from collection failure or inadequate residual amount for testing. Maternal serum and infant cord blood from 258 mother–infant pairs in Nepal and 49 pairs in Seattle were tested using ZEUS IgG enzyme-linked immunosorbent assay (ELISA) kits (Alere, Orlando, FL) for qualitative analysis of rubella IgG and measles IgG. Both measles and rubella kits showed similar reproducibility results in manufacturer trials with 5.8–8.7% coefficient of variation for rubella IgG ELISA and 4.6–9.5% for measles IgG ELISA. Specimens from Nepal were heat-inactivated at 56°C for 30 minutes for a prior assay (Supplemental Appendix).

Data were merged and compared in Stata 13.0 (StataCorp., College Station, TX) and SAS 9.4 (SAS Institute, Cary, NC) using χ^2^ and Fisher’s exact tests. Preterm birth was defined as birth < 37 weeks of gestational age, low birthweight as weight at birth < 2,500 grams.

Participating Nepali mothers, compared with the Seattle cohort, were younger with 225 mothers (87%) less than 30 years of age as compared with 11 Seattle mothers (26.2%; *P* < 0.0001) (Table 1). Nepali mothers were likely to have more children with 167 (66.5%) having between one and three children in the household as compared with 17 (40.5%) in Seattle (*P* = 0.0001). All pregnancies resulted in live births. No preterm or low birthweight infants were observed in Seattle, whereas both were present in Nepal (preterm birth: *N* = 24; 9.3%, *P* = 0.03 and low birthweight: *N* = 44; 17%, *P* = 0.003) consistent with the overall study population.

**Table 1:** Clinical and sociodemographic characteristics of mother–infant pairs in two populations of pregnant women in Seattle, WA and Sarlahi, Nepal

	Seattle (*N* = 49); *n* (%)	Nepal (*N* = 258); *n* (%)	*P* value
Male gender of infant	15 (35.7)	134 (51.9)	0.05*
Maternal age			
< 30	11 (26.2)	225 (87.2)	< **0.0001**
30–35	24 (57.1)	26 (10.1)	–
> 35	7 (16.7)	7 (2.7)	–
Other children in the household (< 15 years)			
0	25 (59.5)	55 (21.9)	0.0001*
1–3	17 (40.5)	167 (66.5)	–
4+	0 (0.0)	29 (11.6)	–
Previous miscarriage	17 (40.5)	10 (7.0)	< **0.0001**
Preterm birth (< 37 weeks)	0 (0.0)	24 (9.3)	0.0329*
Tobacco smoking during pregnancy	0 (0.0)	9 (3.4)	0.5213*
Low birthweight (< 2,500 g)	0 (0.0)	44 (17.1)	0.0026*

Missing values: maternal age = 7, other children in household = 7, miscarriage = 122, gestational age = 7, maternal smoking = 6, birthweight = 10.

* Chi-square test and Fisher’s exact test were used for comparisons between the two site locations. *P* < 0.05 was considered statistically significant (bolded values).

Despite differences in baseline demographics and rubella vaccination status, both populations had high rates of protective IgG antibodies against measles and rubella (Table 2). Two hundred and forty-four (95%) pregnant women in Nepal had protective measles antibody as compared with 44 (92%) in Seattle (*P* = 0.42). Ninety-six percent of infants in both Nepal (*N* = 246) and Seattle (*N* = 43) had protective measles antibody (*P* = 0.75). Ninety-four percent of pregnant women in both Nepal (*N* = 242) and Seattle (*N* = 45) had protective rubella antibody (*P* = 0.23). Altogether, 238 (93%) infants in Nepal had protective rubella antibody as compared with 44 (98%) in Seattle (*P* = 0.12).

**Table 2:** Measles and rubella IgG immune status of mothers and infants in two populations (Seattle, WA, and Sarlahi, Nepal)

	Mothers			Infants		
	Nepal *N* = 258	Seattle *N* = 48	*P* value*	Nepal *N* = 256	Seattle *N* = 45	*P* value*
Measles IgG positive *N* (%)	244 (94.6)	44 (91.7)	0.42	246 (96.1)	43 (95.6)	0.75
Rubella IgG positive *N* (%)	242 (93.8)	45 (93.8)	0.23	238 (93.0)	44 (97.8)	0.12

Missing values: maternal measles = 1, maternal rubella = 1, infant measles = 6, infant rubella = 6. Equivocal values: Nepal: maternal measles = 3, maternal rubella = 0, infant measles = 2, infant rubella = 2. Equivocal values: Seattle: maternal measles = 1, maternal rubella = 1, infant measles = 0, infant rubella = 1.

* Chi-square test. All pregnancies resulted in live birth. Differences in denominator due to failure to collect sample or inadequate residual amount for testing.

Among 23 preterm Nepali infants with antibody results, 8.7% lacked protective measles antibody, compared with 4.7% of full-term infants (*P* = 0.119) and 4.3% of preterm infants lacked protective rubella antibody, as compared with 6.0% of full-term infants (*P* = 0.423). These nonimmune preterm infants matched the serostatus of their mothers.

In two Seattle pairs, mothers lacked protective measles antibody levels, but infant cord blood contained protective levels. Similarly, in two Nepal pairs and one Seattle pair, mothers lacked protective rubella antibody, but infant cord blood contained protective levels. In three Nepal pairs, the mother had protective rubella antibody levels, but infant cord blood did not contain protective rubella IgG levels (Figure 1). All serodiscordant infants were delivered full-term with normal birthweight.

**Figure 1.**
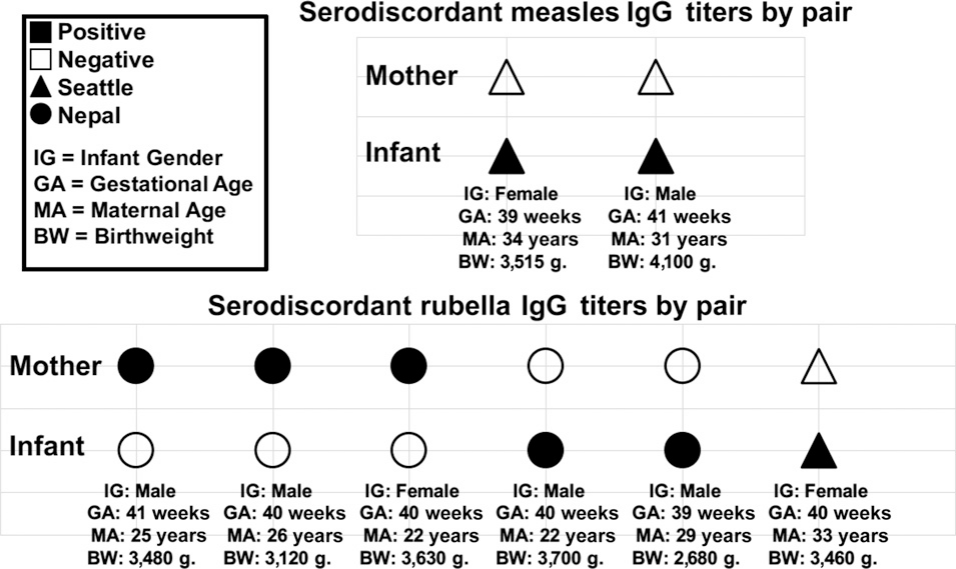
Description of cases of serodiscordance in mother–infant pairs in Nepal and Seattle, WA, by disease-specific antibody serostatus, infant gender, gestational age, maternal , and infant birthweight.

The transfer of measles and rubella IgG between mother–infant pairs in these significantly different populations has not been compared in the literature in the context of recent immunization campaigns. Despite differences in vaccine practices, high levels of measles and rubella IgG immunity were observed in both populations. Maternal rubella immunity in Nepal in this cohort results from natural infection, contrasting with vaccine-induced rubella immunity in Seattle women.

Placental transfer of measles and rubella IgG immunity is critical to protection of infants before vaccination. Most of the maternal antibody is transferred to the fetus by active transport during the third trimester of pregnancy. Higher titers are often observed in infants at birth than in mothers.^15^ Substantial antibody decay before 4 months of age has been well documented, signaling greater risk of infection to infants before they receive vaccine.^16,17^

Preterm birth, maternal inflammation, and autoimmune disease have been identified as possible causes of failure of protective transplacental antibody transfer from the mother to the infant.^16,18,19^ We observed failure of protective antibody transfer of rubella IgG in three full-term Nepali infants. However, the sample size was insufficient to address the effect of gestational age on serostatus in this population. Further quantitative analysis of total maternal IgG could investigate the effect of inflammation and autoimmune disease on measles and rubella antibody transfer.

In addition to sample size, study limitations include missing values as noted (Tables 1 and 2), lack of documented subject vaccine history, and the qualitative nature of the assay. Borderline titers (< 1% of all samples) fell within the equivocal range. It is possible that low positive titers within the reported coefficient of variance were interpreted as negative; however, no serodiscordant pairs fell within this low positive range, most likely demonstrating true serodiscordance.

Serosurveillance studies in tandem with accurate vaccination estimates are vital to monitoring vaccine efficacy and identifying postvaccination trends to ensure adequate vaccine coverage. Inadequate vaccination in either population poses a threat to the high rates of immunity. Infants could become vulnerable to viral infection before the vaccine can be administered. Supplemental vaccination of other groups could be required to protect this high-risk group from disease. As measles and rubella vaccination continues, surveillance must be maintained to ensure high levels of disease-specific antibody, inform progress toward disease elimination in Nepal, and avoid reemergence in the United States.

## Supplementary Material

Supplemental appendix
