# Supplementary material for: Measles and Rubella Seroprevalence in Mother–Infant Pairs in Rural Nepal and the United States: Pre- and Post-Elimination Populations
Source: Am J Trop Med Hyg. 2018 Sep 17;99(5):1342–5. doi: 10.4269/ajtmh.17-0836 (PMC6221218; doi:10.4269/ajtmh.17-0836)
Supplement: Supplementary file 1 [file tpmd170836.SD1.doc]

**Appendix:**

All samples from Nepal were previously heat inactivated for at 56°C for 30 minutes. To model the effect of heat inactivation on the index values of the IgG ELISA assays, sample sera from Seattle were tested in triplicate with one aliquot untreated at room temperature (RT), one aliquot heat inactivated at 37°C for 30 minutes and one aliquot heat inactivated at 56°C for 30 min. The batch of sample sera included known positive and negative controls for both measles and rubella. In this trial, the ELISA IgG index values for room temperature samples had a linear relationship to the index values of heat-inactivated samples (y=1.0555x with R^2^ value = 0.93 for measles IgG, y=1.0248x with R^2^ value = 0.99 for rubella IgG). Two tailed paired t-test between RT and 56°C-inactivated index values yielded p = 0.06 for the rubella heat inactivation trial and p = 0.001 for measles heat inactivation trial.

Further, only a small subset of negative values from untreated samples fell within the equivocal range (0.90 to 1.10) after heat inactivation at 56°C. 3/26 samples in the measles run shifted results in the measles trial and 1/26 shifted results in the rubella trial. Index values of rubella IgG seropositive samples in the Nepal cohort (n = 559) were markedly higher than the equivocal range cut-off values (mean = 4.94, median = 5.18, std. dev. = 1.38). Only two samples fell within the equivocal range.


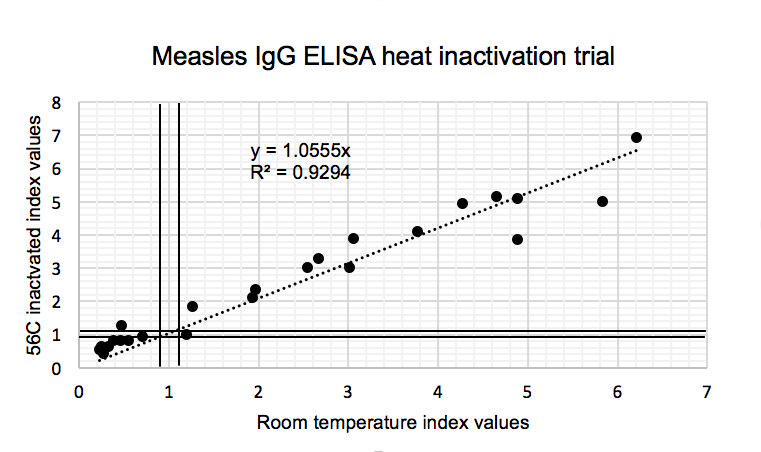


**
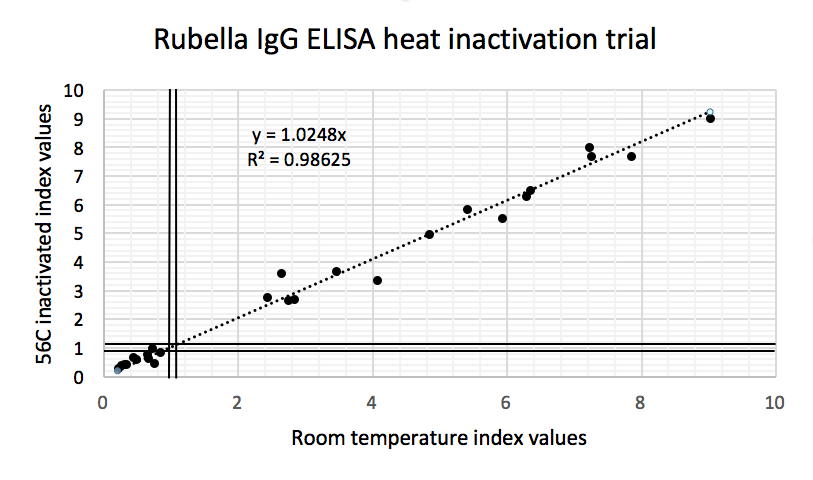
**
